# Cement

**Published:** 1873-04

**Authors:** 


### CEMENT.

Dissolve some glue in warm water, then dissolve some India rubber in spirits of nitre and mix the two, and a cement will be made of great value in a household, for a variety of purposes.

